# Psychosocial burden of recurrent uncomplicated urinary tract infections

**DOI:** 10.3205/id000078

**Published:** 2022-03-24

**Authors:** Kurt G. Naber, José Tirán-Saucedo, Florian M. E. Wagenlehner, RECAP group

**Affiliations:** 1 Department of Urology, Technical University of Munich, Germany; 2 IMIGO/Instituto Mexicano de Infectología Ginecología y Obstetricia, Obstetrics and Gynaecology/Infectious Diseases, Monterrey, México; 3 Clinic for Urology, Pediatric Urology and Andrology, Justus Liebig University, Gießen, Germany

**Keywords:** women, recurrent urinary tract infections, psychosocial impact, quality of life, mental health, sexual function, behavioral modification, immunoactive prophylaxis, antibiotic prophylaxis, treatment

## Abstract

**Introduction:** Urinary tract infections (UTI) are a leading cause of bacterial infections in women. Despite acute treatment, 30–50% of women who have a UTI will experience a recurrence within 6–12 months. In this review, the focus will be on the personal psychosocial impacts of recurrent UTI.

**Methods:** A PubMed/MEDLINE literature search was carried out from 2000 to 2020 in order to identify any recent high-quality meta-analyses or systematic reviews on these topics.

**Results:** One systematic review was found appropriate for this manuscript. Concerning impact on quality of life (QoL) and daily activities, a reduced quality of both intimate and social relationships, self-esteem, and capacity for work was found due to recurrent UTI. Social function was substantially more reduced than physical function. In one study, the greatest reduction overall was in mental role functioning, whereas in another study, mental health reductions were not substantially greater than those of physical health. About one third of women suffered from UTI very often or often after sexual intercourse, and more than half of the patients stated that sexual relations were negatively influenced by UTI. Data from the GESPRIT study suggest that prophylaxis for recurrent UTI is underutilized, because less than 40% of the study population were offered prophylaxis after experiencing three UTI per year, despite all surveyed participants being willing to undertake at least one of the prophylactic measures listed in the survey.

**Conclusions:** Little data on the psychosocial impact of recurrent UTI are available. Therefore, future studies must also incorporate QoL assessments as key outcome measures.

## 1 Introduction

Urinary tract infections (UTI) are a leading cause of bacterial infections in women [1] and amongst the most common bacterial infections in general, with the majority being attributable to *Escherichia coli* [2]. These infections tend to recur, and this tendency increases with each additional infection [3], [4], [5], [6]. The frequency of recurrence varies depending on the type of infection, as well as patient age and gender (Table 1 (Tab. 1)). Despite acute treatment, 30–50% of women who have a UTI will experience a recurrence within 6–12 months [3], [5].

The current European Association of Urology (EAU) guidelines define recurrent urinary tract infections (UTI) as recurrences of uncomplicated and/or complicated UTI with a frequency of at least three UTI in the past year, or two UTI in the last 6 months [7], [8]. Risk factors for recurrent UTI are discussed in depth by Cai [8]. The principal risk factor in sexually active pre-menopausal women is frequency of sex [8], [9]. Other behaviors including use of spermicide, having a new sexual partner within the past year, pre/post-coital voiding habits, delayed voiding habits/periodicity of urination and vaginal douching also affect risk of recurrence [8], [9]. In addition, early onset (<15 years old), family history, body-mass index and urine-voiding disorders all increase risk in younger women [8], [9]. Major risk factors in older women appear to be substantially related to the effects of reduced oestrogen levels and include atrophic vaginitis, cystocoele, increased post-void urine volume and functional status deterioration [10], [11]. Cai et al. have created a nomogram for the calculation of risk of recurrence which has substantial clinical utility [12].

The costs associated with community-acquired UTI overall are significant, amounting to around 1.6 billion USD each year in the United States [13]. Like uncomplicated UTI in general, recurrent UTI have a relatively low cost burden per case. However, the substantial incidence means that a high cost burden remains [14]. In fact, the cost of each episode of recurrence is lower than that of a patient’s first episode. While this may seem like a positive point, it is highly likely that this reduced cost is due to patients working or conducting day-to-day activities despite feeling acutely unwell [2], [14]. Hence, the economic cost is absorbed as a personal burden. It is these personal psychosocial impacts that will be the focus of this review.

## 2 Methods

This content builds upon the discussions of the *RE*current *C*ystitis *A*wareness *P*rogram (RECAP) group (see Acknowledgments section) concerning the personal burden of recurrent UTI. These discussions were conducted as part of a program aimed at raising awareness of unmet needs related to the diagnosis, burden, and management of recurrent cystitis. In addition, a PubMed/MEDLINE search was carried out on literature from 2000 to 2020 using the following terms: (“recurrent UTI” OR “recurrent urinary tract infection”) AND (“anxiety” OR “depression” OR “quality of life”) in order to identify any recent high-quality meta-analyses or systematic reviews as well as other relevant publications. For simplicity, definitions of recurrent UTI will not be mentioned unless they stray from the standard definition used by the EAU and herein.

## 3 Results and discussion

### 3.1 Search results

The relationship between acute symptomatic UTI and reduced quality of life (QoL) has been well established for some years [15]. Yet, in the single systematic review currently available on the subject, Bermingham and Ashe identified no studies specifically investigating the link between recurrent UTI and QoL [15]. We performed an additional search, using the above search terms, for studies published in the 8 years since the Bermingham and Ashe publication. The search revealed 61 new studies. Ten studies were found to be appropriate for this review. Unfortunately, they were not carried out according to standard analytic procedures.

### 3.2 Impact on quality of life and daily activities

As previously noted, recurrent UTI is typically viewed as a condition with low morbidity. However, its effects on QoL can be substantial and multifaceted. Qualitative studies are useful both to shed light on potentially under-reported morbidity and on the substantial burden which seemingly minor symptoms may inflict upon patients. In one such study using data from a large UK Internet self-help forum hosted by a charity supporting people with bladder problems (N=5,994), the authors highlighted patient descriptions of reduced quality of both intimate and social relationships; self-esteem; and capacity for work due to recurrent UTI [16]. Women also frequently described broader systemic disabling symptoms than those typically ascribed to UTI, including flu-like symptoms, spasms, and both back and leg pain [16]. In addition, seemingly mild symptoms such as increased frequency and urgency of urination were discussed in terms of their anxiety-inducing effects and disruption to sleep patterns with the potential to cause persistent fatigue [16]. Contributors discussed equally broad effects of recurrent UTI on day-to-day life, including periods off work with financial and social consequences, and lack of interest in activities beyond work or child care. As stated by one contributor, “I find that this affects every aspect of my life”.

The European GESPRIT (*GE*rmany, *S*witzerland, *P*oland, *R*ussia and *IT*aly) study used a self-administered online survey which assessed course of disease; social and economic burden; disease management and QoL effects (SF-12v2 questionnaire) related to recurrent UTI [17], [18]. The study included adult women who had suffered from recurrent UTI and who were currently affected by an acute UTI or had experienced an episode within the 4 weeks prior to entering the study. At least one episode of recurrence was required to be medically diagnosed, although the acute UTI could be self-diagnosed.

Around half of the participants (51%) were below the US population norm for the physical component summary of the SF-12v2, with the greatest reduction in general health. There were also substantial reductions in role physical (SF-12 category covering day-to-day physical activities such as work, housework, etc.), which decreased to a slightly greater extent than bodily pain compared with the US norm, and in social function suggesting potential reductions in the participants’ ability to carry out day-to-day social and physical activity.

Indirect burden was assessed using days where symptoms limited activities, or the number of days of sick leave due to a UTI. Approximately, three days of sick leave were taken per year across the full study population (2.3 days in Switzerland to 3.9 days in Germany). Limitations to daily activity occurred on approximately 3.5 days per year in the full population (2.6 in Poland to 4.0 days in Russia) [18]. The extent of healthcare utilization can also be used as an indirect measure of disruption to day-to-day activities. The mean number of doctor visits per year was 2.8, ranging from 1.7 visits in Russia to 3.7 visits in Germany (P<0.0001). Only 7% of the participating German women did not have a medical visit at all, whereas 8% reported more than ten medical visits per year. Similar percentages were reported in Switzerland, where 10% of women did not have any medical visit, and 7% had more than ten visits per year [18]. In contrast, 36% of Russian women had no medical visits, while only 3% reported more than ten visits per year. The low number of doctor consultations in Russia could partly be explained by a higher proportion of hospital visits (66% of Russian women) [18]. The majority of women (80.3%) received antibiotics during the study. Those in Germany were most likely to receive a prescription (89.7%), with those in Russia being the least likely (69.8%). In addition to the societal and personal burden associated with the collateral damage to bacterial flora and the potential for resistance, receipt of prescription medicines is also associated with an inherent burden related to interference with day-to-day activities and changes in patients’ beliefs regarding their health and well-being [19]. Recently, researchers in Singapore conducted a study of the effects of recurrent UTI (≥2 documented UTI in the past 12 months) on QoL, enrolling women from a single urological referral center. The recruited women reflected the ethnic breakdown of Singapore (59% Chinese descent, 7% Malay, 9% Indian, and 25% other ethnicities) (N=85) [2]. QoL was assessed using the SF-36, which was adjusted for age and gender in all participants, and for ethnicity in the subset of Chinese, Malay, and Indian participants (n=64), for whom normative data were available.

Recurrent UTI reduced QoL for all the SF-36 physical characteristics, except for physical functioning (P<0.01) (Table 2 (Tab. 2); significance level adjusted for multiple tests, see legend in Table 2 (Tab. 2)). This physical phenotype of reduced physical role function (ability to carry out day-to-day physical activities like work), with unaffected physical function (ability to carry out specific physical functions such are running, climbing stairs, etc.) would be expressed as reduction in the amount, type, or quality of vigorous and moderate daily activities which could be carried out (Table 2 (Tab. 2)). The greatest reduction compared with adjusted normal values was in the bodily pain score, which is perhaps unsurprising, given that participants were assessed while in active consultation for treatment, meaning many patients were likely suffering an acute recurrence during the study (Table 2 (Tab. 2)). Interestingly, social function was substantially more reduced than physical function (Table 2 (Tab. 2)), and the authors speculated that the symptoms of frequency/urgency could be responsible for reduced social function, while the social stigma of UTI may also have been a contributory factor [2].

Ennis et al. [2] also performed a two-sample t-test in order to investigate the relationship between QoL and other demographic and clinical features in the Singapore recurrent UTI population. Chronic constipation and caffeine consumption were found to independently reduce QoL in this population. Both these characteristics have previously been linked to urological disorders, with constipation directly linked to recurrent UTI in children [20]. However, despite the suggested etiological links [21], [22] between these conditions noted by the authors, a χ^2^ test showed no link between chronic constipation and increased infection frequency (χ^2^=2.6274, P=0.105).

### 3.3 Mental health

The QoL measures used in both the above studies also assess several aspects of mental well-being. In the online European GESPRIT survey, SF-12v2 results suggested that women with a history of recurrent UTI and suffering a recent acute episode had more substantially decreased mental health than physical health. While 51% of the population were below the US population norm for the physical-component summary, 78% were below the norm for the mental-health component summary [18]. The GESPRIT study design allowed a comparison between the effect of acute infection and of recurrence. The majority of women who were experiencing an acute infection during completion of the survey reported both lower physical and lower mental component scores compared to the US general norm. Conversely, in women without a current acute infection, a substantial minority still reported worse physical health scores, while the majority were still reporting worse mental-health scores than the US norm [18].

For the mental components of SF-12v2, the most significant reduction overall was in mental role functioning, followed by the previously mentioned social functioning and mental health, which both reduced by a similar extent [18]. With the exception of vitality, all mental-health components were reduced by a similar or greater extent than physical components, and the overall mental score was substantially lower, as mentioned above.

In the Singapore study [2], recurrent UTI reduced QoL across all the SF-36 mental components (Table 2 (Tab. 2)). However, unlike in the GESPRIT study, mental-health reductions were not substantially greater than those of physical health. Again, it is tempting to speculate that the potentially higher incidence of acute infection may have altered the balance of mental and physical health impacts in this population, compared with that of the GESPRIT study.

In a single-center study carried out in Chicago, USA, which recruited adult female patients presenting for initial evaluation of pelvic-floor symptoms, participants with recurrent UTI had higher pre-visit State-Trait Anxiety Inventory (STAI) scores (median, 52; interquartile range [IQR, 50–57) than those without recurrent UTI (median, 40; IQR, 33–50; P=0.02) [23]. The authors noted that there was no association between the degree of symptom distress as measured by Pelvic Floor Distress Inventory and STAI scores. However, given the very small n-number (n=6), we cannot draw any firm conclusions from this study.

Similarly, in a 6-month prospective observational study of prophylaxis against recurrent UTI, further detailed below, 32%, 28%, and 2% of patients (N=575) exhibited mild, moderate, and severe levels of depression, respectively, at baseline as measured by the Hospital Anxiety and Depression scale (HAD). In addition, 30% of patients displayed a high anxious-depressive state [24].

When assessing data from a large (N=30,000) Internet-based, cross-sectional, population-based survey conducted between June 2007 and April 2008 in the USA, UK, and Sweden, Coyne et al. took a novel approach when examining QoL in people with lower-urinary tract disorders by creating subgroups based on symptom clusters [25]. Recurrent UTI was associated with patients with the highest symptom burden (voiding, storage, and post-micturition; P<0.001) and men with this symptom cluster also had higher rates of chronic anxiety and depression than patients in other symptom clusters.

### 3.4 Sexual function

In an already neglected field of research, the effect of recurrent UTI on sexual function is particularly under-researched. In contrast, sex was one of the major topics of discussion identified in the previously mentioned qualitative analysis of an Internet support forum. The well-understood relationship between sex and UTI led to feelings of disgust and fear related to sex, and women reported substantial negative effects on relationships [16].

The GESPRIT study examined the relationship between UTI and sexual intercourse [18]. Of the 90% of women who answered questions related to sexual function (n=1,745), 34% suffered from UTI very often or often after sexual intercourse, with a substantially higher proportion of patients (57%) stating that sexual relations were negatively influenced by UTI. The majority of women (69%) did not use barrier methods of contraception or spermicides, both of which are suggested risk factors for recurrent UTI [9].

### 3.5 Treatment and prophylaxis

Appropriate antibiotic therapy is recommended for acute recurrence, and effective treatment of acute uncomplicated UTI is known to improve QoL [26], [27]. Nevertheless, beyond the well-recognized issues of antibiotic resistance [28], many women with recurrent UTI have a complex relationship with antibiotic therapy. Viewpoints range from concerns regarding overprescribing; lack of efficacy, and short and long-term side effects; to expressions of satisfaction due to the sustained benefits of therapy [16].

Current European guidelines (evidence level) for prophylaxis against recurrent UTI suggest: giving advice on behavioral modifications (weak); use of vaginal estrogen replacement in post-menopausal women (weak); immunoactive prophylaxis to reduce recurrent UTI in all age groups (strong); and use of continuous or post-coital antimicrobial prophylaxis to prevent recurrent UTI when non-antimicrobial interventions have failed (strong) [7]. Data from the GESPRIT study suggest that prophylaxis for recurrent UTI is underutilized. Less than 40% of the study population were offered prophylaxis after experiencing three UTI per year, despite all participants surveyed being willing to undertake at least one of the prophylactic measures listed in the survey [18].

The EAU recommendations for immunoactive prophylaxis are based on data from the oral bacterial lysate therapy OM-89. The effect of OM-89 prophylaxis on mood and QoL were examined in a 6-month prospective observational study conducted in seven countries (Egypt, Germany, Lebanon, Peru, Poland, Portugal, and Switzerland) from 2005–2006, which recruited both adult male and female patients affected by recurrent lower UTI [24]. In total, 575 participants completed the 6-month follow up, 5% of patients did not receive prophylaxis, while most of the remainder (94%) received OM-89 (QD for 90 days), followed by a 3-month treatment-free period.

**Figure 1:**
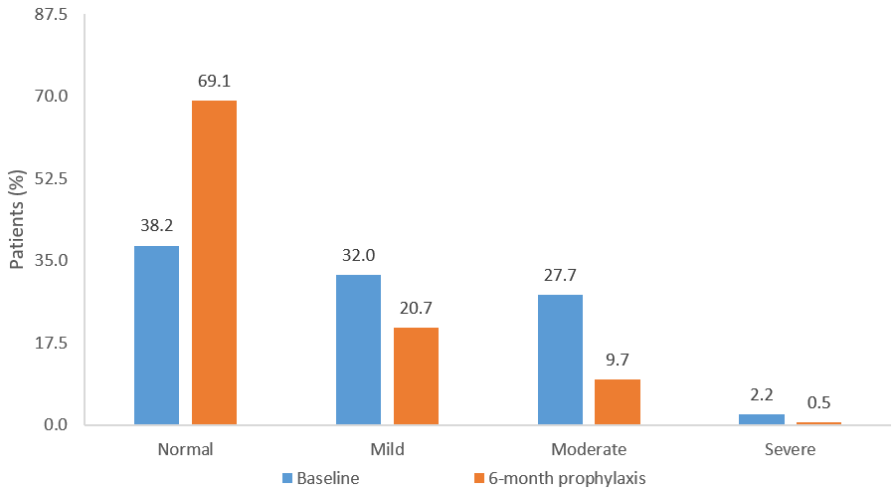
Proportion of recurrent UTI patients with normal to severe anxiety/depression according to Total Hospital Anxiety and Depression score at baseline and following six months of prophylaxis (principally with OM-89), analysis population N=575 [24]

Assessment of mood using the Hospital Anxiety and Depression scale (HAD) showed 32%, 28%, and 2% of patients exhibited mild, moderate, and severe levels of depression, respectively, at baseline. In addition, 30% of patients displayed a high anxious-depressive state [24]. After 6-month prophylaxis, there was a 31% decrease in patients experiencing mild to severe anxiety or depression, and a significant 32% improvement in total HAD score from baseline (P≤0.0001) (Figure 1 (Fig. 1)). The social and functional impact of UTI was measured using the Leicester impact scale, a validated QoL assessment for patients with urinary storage symptoms [29]. Overall, the mean Leicester impact scale score improved by 44% compared with baseline (P≤0.0001). In addition, there was a 33% improvement from baseline in the activity score and a 55% improvement from baseline in the feeling score (both P≤0.0001) following six months of prophylaxis. These changes mirrored the significant decrease of 59% in the number of UTI (P≤0.0001), although a correlation analysis did not reach significance.

Antimicrobial prophylaxis is recommended in cases where non-antimicrobial methods have failed to prevent recurrence [7]. A small (N=12) prospective open-label single-center study conducted in the Netherlands assessed pharmacokinetic, clinical, and QoL outcomes in adult patients with recurrent UTI. Patients were included if they had confirmed infection due to *E. coli* and had received 3 g of prophylactic oral or intravenous fosfomycin every 72 h for at least 14 days. Patients received prophylaxis for between 1 and 75 months, and mean QoL improvement was 2.3 points (10-point scale; P≤0.001) [30].

Hyaluronic acid prophylaxis is not currently recommended in the EAU guidelines, which highlight the need for large-scale trials to assess its benefit. However, a recent single-center retrospective database analysis from an Italian urogynecological center examined the effect of hyaluronic acid prophylaxis in women experiencing post-coital recurrent UTI. Consecutive records from 98 women aged 18–45 years old with a history of recurrent UTI and cystitis after intercourse, but without a current positive urine culture at baseline, were included. Patients received an orally administered combination of hyaluronic acid chondroitin sulfate, curcumin, and quercetin twice daily for the first month, and once daily for the next five consecutive months. Records from women treated with antibiotics for their recurrent UTI within a month of initiating prophylaxis were not included [31].

Following 6 months of prophylaxis, there were improvements in all QoL measures assessed (P≤0.0001), including Pelvic Pain and Urinary Urgency Frequency (PUF) patient-symptom scale [32], SF-36 [33], Female Sexual Function Index (FSFI) [34], and Female Sexual Distress Scale (FSDS) [35]. QoL improvement occurred in conjunction with a low recurrence rate (7%), reduction in positive urine cultures (3.84 vs. 0.35, P<0.0001), and reduction in all UTI symptom categories assessed by the Urinary Tract Infection Symptoms Assessment (UTISA) questionnaire [36].

Similarly, in a small prospective case review study, outcomes of 30 minutes of intravesical sodium hyaluronate prophylaxis were investigated in a mixed cohort of patients with recurrent UTI (n=13) and painful bladder syndrome/interstitial cystitis (n=8). Intravesical treatment was administered weekly for 4 weeks, then once per month thereafter [37]. Mean duration of treatment was 23 weeks in the recurrent UTI group. Mean follow-up was 21 months. Patients showed improvements in bladder pain assessed on a 10-point visual analog scale from 7.8 to 3.4 (P=0.0005), QoL improved from 2.2 to 5.4 (P=0.0207), and daytime frequency decreased from 11.4 to 7.9 (P=0.0354) [37].

Treatment of asymptomatic bacteriuria is not currently recommended for most patient groups. However, the effects of antibiotic prophylaxis on clinical and QoL outcomes in patients with recurrent UTI and asymptomatic bacteriuria have been examined in a single-center randomized prospective Italian study. Women aged 18 to 40 years old attending a sexually-transmitted disease center for treatment of recurrent UTI between 2005 and 2009 were enrolled if they were currently exhibiting asymptomatic bacteriuria (at least 10^5^ colony-forming units [CFU]/mL of uropathogens) [4], [38]. Eligible women were regularly sexually active with a single sexual partner during the last 12 months, and had been treated for at least one symptomatic UTI during the 12 months prior to, but not within one month of, their current episode of asymptomatic bacteriuria. Participants were randomized to receive no treatment for their asymptomatic bacteriuria (n=312) or to be treated with antibiotic therapy according to the local antibiogram with a treatment duration appropriate to the specific antibiotic (n=361). All symptomatic episodes of UTI were treated in both groups during the study. Follow-up visits, including microbiological analysis, were scheduled at 3, 6, and 12 months. QoL was assessed using the Italian version of the Quality of Well-Being Scale [38], [39].

Advantages for untreated patients were present at 6 months, and remained at 12 months for both recurrence (14.7% vs. 73.1%; relative risk [RR]: 3.17; 95% CI: 2.55–3.90; P<0.0001) and QoL (t=134.20; degrees of freedom = 507; SE=0.002; P<0.001) [38]. A Kaplan-Meier curve analysis showed that treated patients had a higher probability of developing recurrence in comparison with untreated patients (RR, 2.14; SE=0.187; P=0.003). It should be noted that more patients were lost to follow-up in the untreated group than the treated group (5% vs. 2%), which may have marginally affected QoL outcomes. Antibiotic therapy was an independent predictor of having a symptomatic UTI in the overall study population (P<0.001; hazard ratio [HR], 3.09; 95% CI, 0.19–4.20), backing up patient perceptions of a negative cycle where infection triggers antibiotic therapy which provides temporary relief but then makes another infection more likely [16], [38].

### 3.6 Further research

Longitudinal studies designed to distinguish between the acute and chronic effects of repeated UTI on patient well-being would be particularly useful. Studies focused on or guided by patient concerns will continue to be of particular import in highlighting or assessing under-recognized issues like improving sexual function in recurrent UTI. We found Flower et al.’s study of an Internet forum particularly enlightening [16]. In the context of increasing resistance to antibiotics, there is a continuing need for research into novel non-antimicrobial prophylaxis, as well as additional well-designed studies for some existing therapies. Such studies are necessary to confirm the positive effect of prophylaxis on QoL, and should be designed with a view to assess whether the effect is a direct consequence of reductions in episodes of recurrence. Research into trial participation shows a clear willingness from women to engage with such trials, driven by the impact of recurrent UTI on day-to-day life [40]. QoL assessment should be incorporated into upcoming trials as a matter of course. Measures such as the Acute Cystitis Symptom Score, a simple symptom scoring tool with integrated QoL assessment, which is recommended in guidelines and available in multiple languages, make assessment of QoL easy to incorporate into study design [41], [42], [43]. The design of current ongoing studies incorporating QoL measures is to be commended [44].

## 4 Conclusions

Little data on the psychosocial impact of recurrent UTI are available. Therefore, future studies must also incorporate QoL assessments as key outcome measures.

## 5 Summary of findings

- Despite acute treatment, 30–50% of women who have a UTI will experience a recurrence within 6–12 months.
- Risk factors for recurrent UTI, although different in sexually active pre-menopausal women as compared to postmenopausal women, need to be analyzed carefully.
- Recurrent UTI have a negative impact on both intimate and social relationships, self-esteem, and capacity for work, whereas social function may be substantially more reduced than physical function.
- About one third of women suffered from UTI often after sexual intercourse.
- More than half of the patients stated that sexual relations were negatively influenced by UTI.
- Prophylaxis for recurrent UTI is underutilized; only less than 40% of the study population were offered some kind of prophylaxis.
- Little data on the psychosocial impact of recurrent UTI are available. Therefore, future studies must also incorporate QoL assessments as key outcome measures.

## Abbreviations

- A/G: Age- and gender-adjusted
- A/G/E: Age-, gender- and ethnicity-adjusted
- BP: Bodily pain
- CFU: Colony-forming units
- EAU: European Association of Urology
- FSDS: Female Sexual Distress Scale
- FSFI: Female Sexual Function Index
- GESPRIT: GErmany, Switzerland, Poland, Russia and ITaly
- GH: General Health perceptions
- HAD: Hospital Anxiety and Depression
- IQR: Interquartile range
- MH: Mental Health
- PF: Physical Functioning
- PUF: Pain and Urinary Urgency Frequency
- QD: Once daily
- QoL: Quality of Life
- RE: Role limitations (Emotional)
- RECAP: REcurrent Cystitis Awareness Program
- RP: Role limitations (Physical)
- SF: Social Functioning
- STAI: State-Trait Anxiety Inventory
- US: United States
- UTI: Urinary tract infection
- UTISA: Urinary Tract Infection Symptoms Assessment
- VT: ViTality (energy)

## Note

This article will also be published as a chapter of the Living Handbook “Urogenital Infections and Inflammations” [45].

## Acknowledgments

The authors would like to thank the other members of the REcurrent Cystitis Awareness Program (RECAP) Board: Yvette Karina Leon-Camacho, Gernod Bonkat, Flavia Rossi, Enrique Ubertazi, Agnaldo Silva Filho de Melo, Tamara Perepanova, Béla Köves, and Tommaso Cai. We would also like to thank Stefania Ballarini and Isabelle Desbrest of OM Pharma for their review and comments. 

## Funding

Funding for editorial and writing support for this article by Ewen Legg, PhD, of Halcyon Medical Writing, was provided by OM Pharma.

## Competing interests

KGN reports within the last 3 years personal fees from Adamed, Apogepha, Aristo, BioMerieux, Bionorica SE, Eumedica, Galenus, Hermes, Immunotek, Janssen, Klosterfrau, Marpinion, Medice, OM Pharma, Roche, Saxonia, and Zambon.

JTS reports within the past 3 years personal fees from MSD, Grünenthal, Bayer, OM Pharma, Qiagen, and Asofarma. Also reports personal fees for advisory board attendance within the past 3 years from MSD, OM Pharma, Bayer, and Grünenthal. 

FW declares personal fees and advisory board attendance and study participation from Achaogen, Bionorica, Klosterfrau Health Group, OM Pharma/Vifor Pharma, and Shionogi. He received personal fees for advisory board attendance from AstraZeneca, Eumedica, Janssen, LeoPharma, MerLion, MSD, Pfizer, RosenPharma, VenatoRx, and GSK.

## Figures and Tables

**Table 1:**
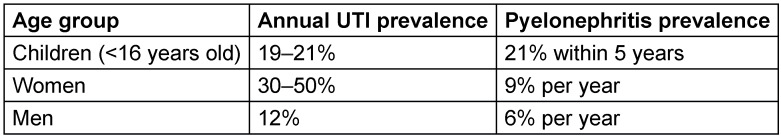
Recurrent episodes per urinary syndrome by sex and age group [5]

**Table 2:**
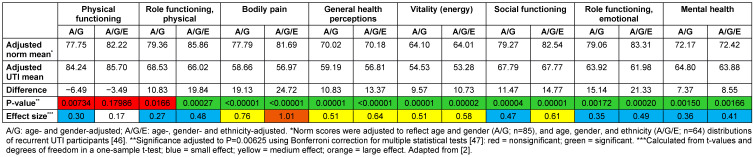
SF-36 multi-item scales in recurrent UTI patients in Singapore compared with age- and gender-adjusted population norms (n=1,349)
